# Effect of a Game-Based Physical Education Program on Physical Fitness and Mental Health in Elementary School Children

**DOI:** 10.3390/ijerph17134883

**Published:** 2020-07-07

**Authors:** Armando Cocca, Francisco Espino Verdugo, Luis Tomás Ródenas Cuenca, Michaela Cocca

**Affiliations:** 1 Department of Sport Science, University of Innsbruck, 6020 Innsbruck, Tirol, Austria; 2 School of Sports Organization, Autonomous University of Nuevo Leon, San Nicolás de los Garza 66455, Mexico; espinopako@hotmail.com (F.E.V.); luistorc23@hotmail.com (L.T.R.C.); michaelacocca@gmail.com (M.C.)

**Keywords:** physical education, children, physical fitness, psychological health, games, exercise training

## Abstract

Promotion of healthy active behaviors should start from early ages, as behaviors learned in youth are more likely to endure. A fundamental body of research in this field focuses on the implementation of programs within physical education (PE), thanks to its favorable characteristics. However, traditional PE based on exercise training and controlling styles seems to have weaker association with students’ health benefits. For this reason, the aim of this study was to assess the effects of a game-based PE program on physical fitness and psychological health in schoolchildren aged 10 to 12 years old. A total of 252 students were distributed in experimental (EG, games-centered activities) and control (CG, traditional exercise training activities) groups. The program lasted 6 months. Health-related physical fitness components, psychological wellbeing, self-esteem, stress, and anxiety were assessed before and after the treatment. Both groups increased physical fitness at post-test; however, cardiorespiratory fitness did not improve. No differences were found between the groups at post-test. Our results show that games may be as effective as traditional training methods; yet, they suggest that PE alone may be insufficient for obtaining substantive benefits in cardiorespiratory fitness, regardless of the type of task presented.

## 1. Introduction

Promotion of health is a necessary action that should be carried out at all ages, as it may trigger benefits both in terms of disease risk reduction and prevention, regardless of the phase of life [1,2]. Such benefits become even more evident if health is fostered from youth [3]. Although all variables involved in the enhancement of health should be taken care of, health-related physical fitness for the physical domain, and self-esteem, psychological wellbeing, anxiety, and stress for the psychological domain, seem to play a key role in youth’s proper growth. Health-related physical fitness comprises cardiorespiratory fitness, i.e., the ability to perform with the whole body for a prolonged period; flexibility, or the range of motion of a joint or group of joints; muscular strength, which indicates the maximum amount of force that a muscle can exert against any resistance on a short time; and muscular endurance, describing the ability of a muscle to sustain several contractions over a prolonged time [4]. Evidence of the value of physical fitness for youth’s health is shown in several studies associating it with, among others, better cardiovascular function [5], movement skills [6], or weight status and obesity [7,8]. Regarding the psychological domain of health, psychological wellbeing is defined as the combination of positive effects and optimal social and personal efficiency [9]; self-esteem represents confidence in and respect of oneself; anxiety is characterized by a feeling of unease about situations with uncertain outcome; while stress is a psychophysical response when facing demanding or adverse environmental conditions. All of these variables seem to be associated with youth’s health levels, although in different ways. For instance, authors suggest that psychological wellbeing, social inclusion, and social support are positively correlated [10]. Similar positive interactions are found with peer relationships [11]; self-esteem seems to play a role in preserving mental health [12], as well as in modulating individuals’ behaviors [13]; childhood anxiety has been linked with increased risk of depression [14] and higher emotional imbalance [15]; finally, findings from previous research highlight a direct association between childhood depression and stress [16], leading to higher risk of the onset of eating disorders and obesity [17].

Authors agree that building active habits from early ages represents a leading strategy for the development of psychophysical health, thanks to their positive effects not only in the short term, but also over time. The relation between youth’s physical activity (PA) habits and adulthood health has been thoroughly examined in previous literature. For instance, Appelqvist-Schmidlechner et al. [18] highlight that engaging in sports in childhood is associated with lower risk of developing mental health issues in adulthood. Ekblom-Bak et al. [19] found that kids who participated in both in- and out-of-school exercise showed reduced incidence of metabolic diseases later in life, as well as higher engagement in healthy active behaviors, and better fitness. The relationship between physical fitness and PA levels is supported in previous research as well [20]. A study tracking a sample from the age of nine until adulthood established a relation between higher muscle strength at early ages with reduced risk of suffering from chronic diseases later in life [21]. Opposed to that, sedentary behaviors and inactivity have been linked with poor health outcomes in adulthood [21,22,23]. Thus, it is clear that youngsters with active habits may not only enjoy higher levels of physical and mental health at once, but also have more chances to carry these habits into adulthood and benefit from their positive effects at later stages of their life.

For this reason, governments, unions, and health associations have established various guidelines and strategies in order to encourage active behaviors among youth, empowering active schools being one of the most essential [24]. This is due to favorable characteristics of the school context; for instance, schools are safe and well-known places for students, as they spend several hours of the day in school facilities. Also, physical education (PE), especially when successfully planned, provides more chances for everybody to be active compared to out-of-school sports facilities, which are subjected to other potential limitations [25]. The benefits of proper PE on health are pointed out in a vast body of literature, which outlines PE effect on increased physical fitness [26], proper diet and overall PA levels [27], or improved mental health [28]. However, the positive influence of PE on health is mediated by several factors, among them teachers’ style and content choices. In fact, more traditional teachers’ approaches to PE, by means of which teachers tend to employ controlling strategies and strict exercise routines, have been linked to lower perception of quality of life, as well as students’ frustration of their basic psychological needs [29]. There is evidence that such traditional, less or no-game-based instruction styles, while still largely used, lead to lower, null, or negative effects of PE on health-related variables [30,31,32]. Opposed to that, successful PE models seem to depend on a more game-based approach, as some authors have pointed out in previous works. For instance, Dominguez and del Campo [33] compared the Sport Education model with a traditional model of PE, stressing the importance of experimenting over repetition in order to internalize learnings in this setting. Physical fitness was the main focus of the work of Lavrin et al. [34], which contrasted a game-based program with the traditional one. At the end of the semester of implementation, students in the game-based program showed higher fitness compared to their peers in the traditional group. These findings are confirmed by Ceballos Gurrola et al. [35], who carried out a ball-game program with primary school children, obtaining significant improvements in the metabolic profile of the students in the experimental group compared to those in the control group.

It is evident that games may represent an important factor for triggering the health benefits of PE [36]. Therefore, for all that is stated above, the aim of this study was to assess the effect of a game-based physical fitness program on health-related physical fitness and psychological health variables in schoolchildren from primary education.

## 2. Materials and Methods

### 2.1. Study Design

The study was developed following a quasi-experimental design with pre- and post-measurements. Quasi-experiments are defined as studies in which participants are not assigned randomly to either experimental or control groups; instead, due to ethical or practical reasons, pre-established groups are maintained during the carrying out of the experiment [37].

### 2.2. Participants

The first step in the selection of the sample consisted in obtaining school administrators’ consent to take part in the study. Once the list of participating educational centers was completed, students from them were recruited based on the following criteria: (a) students were enrolled in 5th or 6th grade, (b) students did not have any diagnosed issue, (c) students participated regularly in PE classes, and (d) students’ parents had signed an official consent form previously distributed by the researchers. The original sample was composed of 486 primary school students. Successively, we decided to consider for the analysis only those students not enrolled in formal out-of-school PA activities, so as to control for their potential effects over fitness and mental health. A total of 252 children (133 boys, 119 girls) aged 10 to 12 years old from four primary schools were hence selected for the study. Existing groups (classes) were preserved. Schools were treated as conglomerates and randomly assigned to the experimental or control protocol. At the end of the intervention, 23 participants were excluded from the analysis due to missing data (22) or due to being detected as outliers (1). Therefore, the final sample was composed of 229 children (125 boys, 104 girls; average age = 10.24 ± 0.50). Detailed information on the experimental and control subsamples is given in Table 1.

### 2.3. Instruments

#### 2.3.1. Physical Fitness

Tests from the Eurofit test battery [38] were used for assessing health-related physical fitness. This battery includes the following tests related to health: (a) Sit-and-reach is used to assess flexibility of the lower back. As suggested in the Eurofit manual, the measure at feet level is set at 15 cm. (b) Shuttle-run test is used for assessing cardiorespiratory fitness. The VO_2peak_ is calculated based on the speed at the last completed stage [39] and using a formula for children proposed in previous work [40]. (c) Handgrip strength is tested by means of a Jamar hydraulic hand dynamometer, which delivers an outcome in kilograms and grams. (d) The 30 s sit-up test consists of attempting to complete as many repetitions of sit-up abdominals as possible in 30 s. The procedures and features for each test in this battery are thoroughly described in the corresponding manual [38].

#### 2.3.2. Psychological Health

The following instruments were used in order to assess the different variables included in psychological health: (a) Psychological Wellbeing Questionnaire (PWBQ) [41], composed of 10 items with Likert scale from 1 (never) to 4 (always) and scores obtained by summing the individual answers, which can range from 10 to 40. In our sample, the structure of the questionnaire showed good validity (χ^2^/gl = 1.802; NFI = 0.953; CFI = 0.967; RMSEA = 0.045). (b) Rosenberg self-esteem scale (RSES) [42], whose original version consists of 10 items. Based on previous literature on children’s questionnaire procedures [43] and considering the two-factor (negative and positive) structure of RSES [44], only the five positive items were used. The instrument employs a Likert scale ranging from 1 (strongly disagree) to 4 (strongly agree), and results are obtained by summing each individual item score. The instrument showed good validity indexes in our sample (χ^2^/gl = 1.299; NFI = 0.995; CFI = 0.998; RMSEA = 0.027). (c) Beck Anxiety Inventory for youth [45], consisting of 20 items with answers ranging from 0 (never) to 3 (always). The validity indexes for our sample were good (χ^2^/gl = 2.75; NFI = 0.963; CFI = 0.977; RMSEA = 0.053). (d) Stress in Children Questionnaire [46], whose language-validated version [47] contains 16 items with Likert Scale from 0 (none) to 3 (always). Higher total scores (sum of items’ answers) indicate higher level of stress. The indexes of goodness-of-fit found in our sample (χ^2^/gl = 1.133; NFI = 0.982; CFI = 0.986; RMSEA = 0.0194) denote good reliability of the instrument.

### 2.4. Intervention Program

The intervention program consisted of focusing the PE classes on game-based activities rather than traditional fitness training. The large majority of the existing game-focused programs implemented in schools predominantly propose sports, or modified sports [33,34,35]; moreover, they mostly examine cardiorespiratory fitness as the main dependent variable, due to its strong relation to health [48]. Our program only marginally included sports-related games, so as to avoid the influence of perceived competence on exercise behaviors [49]. In fact, perceived competence is linked to previous experience; while most sports used in PE are well known by students, new exercise situations may pose a stimulating challenge to everybody. All the activities proposed aimed at increasing fitness through team or individual challenges, modified ball games, target, invasion, cooperation, and skill games, as well as chasing–fleeing and inquiry games. The games were created using innovative pedagogical instructional models, such as inquiry-based learning, cooperative learning, and peer teaching, as the theoretical–practical foundation [50], and presented with an autonomy-focused, student-centered style of instruction. A fundamental rule set for each game was that no student could be excluded from the activity in any moment, so to allow everybody to be active for their whole duration. Each PE session of the program was split into two halves of approximately 15 min each. The first half comprised games implying prolonged aerobic effort, whereas the second focused on games developing basic motor skills, such as jumping, tossing, balance, or catching. A short warm-up at the beginning and a final cooling down completed each session. On the other hand, the control group carried out the traditional PE activities planned for the semester, which targeted the development of physical fitness through training methods based on sets and repetitions of running, own body weight, and/or resistance band exercises. In Mexico, where the study was carried out, traditional training programs in PE are commonly presented by teachers using predominantly a controlling style of instruction, based on imitation and reduced student autonomy [51].

### 2.5. Procedure

This study received the ethical approval of the Ministry of Education of Mexico (reference number: DCA/103.5/16/10510). During the semester prior to implementation of the program, researchers and specialists in PE carried out theoretical and practical workshop sessions with the PE teachers whose classes were selected for the EG. These workshops aimed at providing those teachers with tools and strategies for putting the games into practice with proper methodology, resources preparation, and a more student-centered attitude that would promote pupils’ autonomy and positive in-class motivational climate, so for the features of the intervention to be preserved. In addition, teachers had the possibility to try and familiarize themselves with the program during a two-month pilot implementation with volunteers from the research institution. In this pilot period, researchers and teachers discussed and solved any doubt, question, or concern that could arise. The teachers delivering the control protocol differed from those in the game-based program, and therefore, did not take part in the workshops. This was made to ensure that the CG classes would run in a manner considered usual for the context of the study.

Measurement sessions were carried out in the first and last week of the semester, during the regular schedule of the PE classes and upon permission of the PE teachers. Respecting the weekly calendar of each school involved, physical fitness assessment was performed in two separate sessions both at pre- and post-test. In the first session, all Eurofit and anthropometric tests were delivered, except for the cardiorespiratory assessment. The second session was exclusively used for the shuttle test, due to the fact that it requires a longer time and major effort from the participants. Psychological assessment was also split in two sessions; participants completed the questionnaires before the start of each testing session.

The research took place at the facilities of four primary education schools located in the urban area of General Escobedo, Monterrey, Mexico. The intervention was carried out during a whole winter semester (January to June), maintaining the frequency and duration of regular PE classes as established by the Ministry of Education of Mexico, which consists of two 45-min weekly sessions. During the intervention period, the System for Observing Fitness Instruction Time (SOFIT) [52] was used by trained researchers in order to monitor students’ PA levels in both EG and CG [53]. Once a week, four researchers observed one randomly selected class from each protocol and filled in the observation sheet, which was successively processed according to the SOFIT manual [54].

### 2.6. Statistical Analysis

For the data cleaning, descriptive characteristics and frequencies of each variable were examined, as well as statistical procedures of standardized z scores and Mahalanobis D^2^ were run for the detection of potential outliers. Students’ *t*-tests for paired samples were performed to evaluate intragroup differences between pre- and post-test. Intergroup differences at post-test were assessed by means of analyses of covariance (ANCOVAs) per each dependent variable separately, using the outcomes from pre-test as the covariates. This was necessary due to the fact that experimental and control groups were not created randomly. Finally, effect size was calculated by means of Cohen’s d, with the conventions small (0.2), medium (0.5), and large (0.8) [55].

## 3. Results

Participants in both the traditional and game-based programs showed predominantly moderate intensity levels of PA (64.4% and 59.7%, respectively), never reaching vigorous levels of intensity.

For participants in the EG, significant differences at post-test were found in flexibility (*p* < 0.001), handgrip strength (*p* < 0.001), and stress (*p* = 0.027). No significant differences were found for the other observed variables. Regarding CG students, flexibility (*p* < 0.001), handgrip strength (*p* < 0.001), and abdominal strength (*p* = 0.011) obtained significantly higher scores at post-test. A summary of the pre- and post-test comparisons for the EG and CG are shown in Table 2.

ANCOVAs for each observed variable were run to examine differences between EG and CG at post-test, using pre-test scores as covariates. Findings show no significant differences in any of the variables included in the study, except for handgrip strength in which CG participants obtained higher mean scores than those in EG (*p* = 0.001; η = 0.104). Table 3 presents the detailed results from the ANCOVAs.

## 4. Discussion

The aim of this study was to provide scientific knowledge on the effect of a non-sport, game-based physical education program on physical fitness and mental health in elementary school children. The program lasted for six months and was based on two 45 min classes per week as stipulated within the school’s planning.

### 4.1. Physical Fitness

In our study, both EG and CG participants showed improvements in physical fitness at post-test, with no significant differences between the two groups. This is in line with the results of other authors [26], who highlight that PE can have a strong impact on fitness when carried out properly. While traditional methods of teaching, as well as the selection of less fun activities, have been associated with potential detrimental outcomes in both youth’s psychological and physical health [29,30,31,32], some authors suggest that the effects of teaching styles on students’ outcomes may be mediated by other social and demographic factors [56]. In the specific context of our study, controlling styles and stricter approaches to exercise are common in the area of PE [51]; therefore, it is possible that, being used to such approaches, participants in the CG were less affected by them, and more willing to engage in the activities. Additionally, it is suggested that adolescents with a strong and positive mindset may be able to deal with such teaching styles in a better way, reducing or nullifying their negative effects [57]. In our sample, students from both EG and CG showed very high levels of psychological wellbeing and self-esteem, at the same time as they had very low stress and anxiety. Thanks to that, it is possible that coping with less autonomous approaches and less enjoyable activities was easier for them. Three additional points must be considered here. First, due to the design employed in the study, the influence of maturation on the changes in physical fitness could not be fully controlled. Therefore, this variable may have played a role in the increase of certain physical fitness components in this study. At this stage of life, as well as in adolescence, maturation is known to be a potential influencing factor for PA and fitness [58]. Secondly, the improvements in both groups might have also been determined by the fact that all participants, regardless of the assigned group, had very low fitness levels at pre-test. In fact, it is known that fitness improvements are easier to obtain when the initial fitness level is low [59]. Nonetheless, given that participants were not involved in any out-of-school PA, our findings suggest that two 45-min PE sessions per week are sufficient to provoke significant fitness improvements, regardless of methods and contents proposed by the teachers. Finally, while statistically significant differences were found in handgrip strength, they cannot be considered truly meaningful due to the low effect size.

Cardiorespiratory fitness was the only variable not showing improvements in any of the two groups at the end of the program. Our findings differ from those of previous literature, which support the positive effect of programs of different duration on cardiorespiratory fitness. For instance, Mayorga-Vega et al. [60] employed a circuit training program in two 60-min PE classes per week for eight weeks, showing a significant increase in cardiorespiratory fitness at the end of the experimental period, which did not drop after four weeks of detraining. However, the reported effective class time (40 min) was longer than that in our study, where total allocated time for PE was 45 min, portions of which were used for preparation, warm-up, cooling down, and transfer from/to class. Also, the researchers did not control for out-of-school PA; hence, participation in external activities may have amplified their results. A study from Lopez et al. [61] found that cardiorespiratory fitness, along with fat-mass percentage, improved significantly at the end of a 10-month PE-based program. In line with this study, an eight-month small-sided soccer intervention implemented in high school was found to have a significant impact on cardiorespiratory fitness and other health-related fitness components [62]. Both studies had longer duration than ours, did not control for out-of-school activity, and made use not only of the time allocated officially for PE, but also of recess times. Hence, duration and frequency of the activities seem to be important factors specific to this fitness component. This is in agreement with general exercise and health guidelines released by several international associations, which underline the need for youth to perform aerobic activities at moderate-to-vigorous intensity for at least 60 min per day in most days of the week in order to obtain concrete benefits in terms of fitness and general health [63]. Based on these guidelines, our results can be explained through the study of Vilchez and Ruiz [64], which focused on active habits in the same population as in our work; the authors found a high level of PA drop-out in youth, at the same time as those engaging in some kind of physical exercise only performed it at low intensity. While the observed intensity level in our study was mostly moderate, it never reached the vigorous one. According to McKenzie [54], only the highest observed PA code of SOFIT may fully represent intensities of exercise associated with health and fitness benefits.

### 4.2. Psychological Health

Both EG and CG showed very positive psychological condition at pre-test, which did not change after the intervention. We may understand these results as an extension of the exercise theory according to which the rate of improvement of a certain capacity lowers when its initial level is high, making it more difficult to find strategies and obtain significant positive changes [59]. Participants’ psychological health was already excellent at pre-test, as they reported very high psychological wellbeing and self-esteem, at the same time as they scored very low in anxiety and stress. Therefore, it is logical to assume that increasing those levels through a twice-weekly session PE program would be challenging, even with the use of enjoyment-promoting activities, such as games. Additionally, psychological factors may be influenced by other factors surrounding youth’s life. For instance, Zemp et al. [65] suggest that children’s psychological health may be influenced by the type of relation between the parents; parental emotional availability and peer relationships are also attributed to an essential role in the construction of a positive mindset [66]. Furthermore, some authors propose that even physical pathologies may play a role in it [67]. While we did not have any control over external events like those mentioned above, they are just some of several social and personal occurrences that may have contributed to participants’ psychological condition before, during, and after the intervention. Support to this conjecture may be found in the specific social culture in which the study was developed. Mexican culture is considered to have very high familism and family values [68], as well as high amounts of time spent within the family [69]. In studies carried out on Mexican populations, these variables seem to be associated with youth’s psychological adjustments, at the same time as they reduce the risk of developing depression symptoms [69]. In general, Mexican familism positively influences psychological wellbeing at different age ranges [68]. Marsiglia et al. [70] add that familism and family cohesion in people of Mexican origin serve as protective factors against behavioral problems in adolescents. A final consideration should be done regarding stress, which, despite being already very low at pre-test, was significantly lowered in the EG after the intervention. While we need to take our findings carefully, they support the positive effect of participating in exercise games on the improvement of mental health, and specifically on stress reduction. In fact, lower levels of stress were found in students after the implementation of team sport and dance activities [71]. Active games also seem to have a role in the reduction of stress and anxiety among children with psychological issues [72] and alienation experiences [73], and even in adults [74]. In line with these and our study, Yogman [75] confirms that games are effective antidotes to reduce youth’s aggression and uncontrolled emotion generated by toxic stress.

## 5. Limitations

Among the limitations of this study, we list the following: (a) The design is quasi-experimental and the groups could not be created randomly, opening potential issues related to selection and maturation biases [76]. Due to organizational reasons, it was not possible in our study to form random groups, as class groups had to be maintained. (b) The intervention program was of limited duration. Studies in the same field suggest that impactful results may be obtained with longer implementations and/or higher weekly frequency [60,61,62]. In our case, respecting teachers’ original plans and schools’ official schedules was required. Therefore, this led us to focus the intervention on a six month span. (c) Several potential noise variables could have contributed to the results, among them social and personal events on psychological health. It would be interesting to study the effect of games on students’ perceived in-class motivational climate and enjoyment of PE. In fact, given the particular characteristics of games, they might have an impact on variables that promote daily active habits and mediate the intention to participate in out-of-school PA, therefore, contributing in achieving the amount of exercise recommended for health [77].

## 6. Conclusions

Although some physical condition components were improved, our study suggests that overall levels of health-related physical fitness remain low, insufficient for obtaining health improvements based on the guidelines provided by the World Health Organization (WHO) [63]. In particular, two 45 min sessions per week, regardless of their fun- or training-specific focus, seem to be insufficient to contribute significantly on cardiorespiratory fitness, especially if we take into account that PA intensity was never higher than moderate. This should be addressed through an increase in frequency and duration of PE classes, or through more comprehensive in-school PA programs involving not only PE, but also taking advantage of recess and breaks. Finally, game-based activities appear to have similar impact as traditional approaches in our sample. Therefore, they may be used together or in place of more strict exercise routines for the improvement of physical fitness.

## Figures and Tables

**Table 1:** Descriptive characteristics of the participants.

Protocol	Gender	N	Age		Height (cm)		Weight (kg)	
			Mean	SD	Mean	SD	Mean	SD
Experimental	M	51	10.20	0.50	139.81	7.50	39.97	11.82
	F	51	10.31	0.51	140.65	7.35	41.74	9.61
	Total	102	10.25	0.50	140.17	7.40	40.73	10.89
Control	M	74	10.27	0.53	139.45	5.29	39.07	9.53
	F	53	10.17	0.43	142.73	7.97	41.29	13.32
	Total	127	10.23	0.49	140.94	6.80	40.08	11.38

Note: M = male; F = female; SD = standard deviation.

**Table 2:** Results of the paired pre- and post-test comparison for the experimental and control group.

Variable		Pre-Test (Mean ± SD)	Post-Test (Mean ± SD)	*p*	η
VO_2peak_ (mL × min × kg^−1^)	EG	42.29 ± 3.17	42.32 ± 5.78	0.980	
	CG	43.82 ± 4.74 *	42.96 ± 5.10	0.065	
Flexibility (cm)	EG	14.53 ± 7.64	23.80 ± 6.37	<0.001	>1
	CG	16.16 ± 5.68 *	25.39 ± 7.31	<0.001	>1
Abdominals (n)	EG	13.84 ± 6.27 *	13.12 ± 5.97	0.317	
	CG	12.13 ± 5.08	13.63 ± 4.99	0.011	0.36
Handgrip (kg)	EG	16.50 ± 5.12 *	18.40 ± 4.87	<0.001	0.56
	CG	14.15 ± 3.91	21.56 ± 8.71	<0.001	0.83
PWB	EG	32.86 ± 6.68	32.94 ± 6.45	0.906	
	CG	35.28 ± 4.86	33.85 ± 5.97	0.073	
Self-esteem	EG	14.44 ± 4.00	15.16 ± 3.64	0.269	
	CG	15.58 ± 3.61	15.27 ± 3.16	0.537	
Anxiety	EG	12.24 ± 10.48	13.91 ± 9.41	0.102	
	CG	14.36 ± 13.47	14.19 ± 12.29	0.898	
Stress	EG	20.54 ± 5.44	18.54 ± 6.25	0.027	0.32
	CG	19.62 ± 6.92	19.09 ± 6.43	0.543	

Note: EG = experimental group; CG = control group; SD = standard deviation; PWB = psychological wellbeing; * difference between EG and CG at pre-test < 0.05.

**Table 3:** Results of the univariate analysis of covariance.

Variable	EG (EMM ± SD)	CG (EMM ± SD)	F	*p*	η
VO_2peak_ (mL × min × kg^−1^)	43.13 ± 0.78	42.54 ± 0.563	0.367	0.546	
Flexibility (cm)	24.32 ± 0.80	24.91 ± 0.762	0.290	0.591	
Abdominals (n)	12.54 ± 0.59	14.15 ± 0.561	3.856	0.052	
Handgrip (kg)	17.66 ± 0.95	22.25 ± 0.914	11.710	0.001	0.104
PWB	33.76 ± 0.702	33.08 ± 0.682	0.480	0.490	
Self-esteem	15.35 ± 0.454	15.09 ± 0.445	0.166	0.685	
Anxiety	14.63 ± 1.06	13.50 ± 1.04	0.585	0.446	
Stress	18.27 ± 0.777	19.33 ± 0.755	0.900	0.345	

Note: EG = experimental group; CG = control group; EMM = estimated marginal means; SD = standard deviation; PWB = psychological wellbeing.
